# Editorial for the *IJMS* Special Issue on Molecular Pathogenesis and Molecular Therapy of Lymphoid Malignancies

**DOI:** 10.3390/ijms25010557

**Published:** 2023-12-31

**Authors:** Alexandria P. Eiken, Dalia El-Gamal

**Affiliations:** Eppley Institute for Research in Cancer and Allied Diseases, Fred and Pamela Buffett Cancer Center, University of Nebraska Medical Center, Omaha, NE 68198, USA; alexandria.eiken@unmc.edu

In the era of targeted therapies, researchers have aimed to uncover the molecular drivers of malignant pathogenesis in lymphoid malignancies in an endeavor to develop effective therapeutic strategies. Lymphoid malignancies are neoplastic disorders of the immune system that are classified in terms of cell lineage (B-cell, T-cell, and NK-cell), cell maturity (immature vs. mature), and clinical behavior (indolent vs. aggressive) [1,2]. The majority of B-cell-derived lymphoid malignancies are dependent on B-cell receptors (BCRs) and NF-κB signaling, the overexpression of oncogenic (e.g., MYC) and anti-apoptotic (e.g., MCL1, BCL2, BCL-xL) proteins [3]. Chemotherapies previously dominated the catalogue of available therapeutic options; however, in recent years, targeted therapies have taken the lead, especially in the setting of relapsed/refractory (R/R) disease. Preferred strategies for patients with B-cell malignancies consist of anti-CD20 monoclonal antibodies (e.g., rituximab), targeted agents against B-cell receptor (BCR) kinases such as Bruton’s tyrosine kinase (BTK) and phosphatidylinositol 3-kinase (PI3K) inhibitors, or against the anti-apoptotic protein BCL-2 (venetoclax), used alone or in combination [3]. Cellular therapies, such as CD19-directed chimeric antigen receptor T-cells (CAR T-cells), are commercially approved for diffuse large B-cell lymphoma (DLBCL), B-acute lymphoblastic leukemia, and multiple myeloma (MM). Clinical trials for CAR T-cell therapies are open and in development for patients with R/R chronic lymphocytic leukemia (CLL) and classical Hodgkin lymphoma (cHL). This Special Issue emphasizes the complex pathogenesis of lymphoid malignancies, the role of the gut microbiome on disease progression and immunotherapies, and the characterization of novel targeted therapies.

In CLL and DLBCL, BCR signaling is a major driver of malignant B-cell growth and survival. Ibrutinib, acalabrutinib, zanubrutinib (BTK inhibitors) and venetoclax (BCL2 inhibitor) have revolutionized treatment strategies for CLL patients [4]. Although the class of PI3K inhibitors has a diminishing clinical role in B-cell malignancies, idelalisib remains approved for CLL. Despite their efficacy, CLL patients still become R/R to these targeted therapies, highlighting a need for targeting multiple biologic facets of the disease. Epigenetic modifiers like the chromatin reader, BRD4, are overexpressed in lymphoid malignancies and contribute to higher expression of oncogenic proteins and disease-relevant factors such as BCR kinases and oncogenic drivers such as TCL1 and MYC [5]. In this Special Issue, Smith et al. [3] describe a novel triple inhibitor (SRX3305) that targets BCR signaling (BTK and PI3K) and epigenetic modifiers (BRD4) in CLL cells by covalently binding to its targets. SRX3305 inhibited the activation-induced proliferation of primary CLL cells in vitro and effectively blocked survival signals mediated by the tumor microenvironment (TME), including stromal cell support. Furthermore, SRX3305 blocked CLL cell migration toward CXCL-12 and CXCL-13, vital chemokines involved in CLL cell homing and retention in TME niches. Remarkably, SRX3305 induced apoptosis in ibrutinib-resistant cells, lending support for continued clinical development of SRX3305 for CLL patients who relapse on ibrutinib. Overall, the authors provide strong preclinical evidence for the development of a triple-action inhibitor for B-cell malignancies.

Immunomodulatory agents (IMiDs), proteasome inhibitors, and monoclonal antibodies have greatly improved survival for MM patients. However, the overall five-year survival remains dismal at 55% [6], highlighting the continued need for novel therapies targeting the unique pathophysiology of MM. Accordingly, Haney et al. [7] discuss the therapeutic potential of targeting the isoprenoid biosynthetic pathway (IBP) to inhibit MAPK signaling and MM cell proliferation. Ras small GTPases (i.e., Ras, Rho, Rab) control cellular processes such as proliferation, gene expression, cell survival and differentiation within MM cells. The activity of Ras small GTPase proteins is regulated by their prenylation; thus, the inhibition of the IBP leads to an accumulation of unprenylated Ras proteins. Statins, nitrogenous bisphosphonates (NBPs), GGTase inhibitors, and GGDPS inhibitors are being evaluated in relation to MM as possible targeted therapies for the IBP as they have shown impressive preclinical effects. Statins (e.g., lovastatin and simvastatin) inhibit HMG-CoA reductase, the gatekeeper to the IBP, which effectively lowers cholesterol levels and induces apoptosis in MM cells. Additionally, statins have been shown to induce the unfolded protein response (UPR) in MM cells through the disruption of light chain trafficking [7]. Since MM cells are constantly producing and secreting monoclonal proteins, they have a lower threshold for the induction of the pro-apoptotic arm of the UPR. Recent studies have shown that combinations of bortezomib, a proteasome inhibitor, with statin, are synergistic and enhance the induction of UPR. NBPs (e.g., zoledronic acid) target the next enzyme in the IBP, farnesyl diphosphate synthase, and are potent inhibitors of bone resorption and bone remodeling, making them useful for treating MM bone disease. Lastly, GGDPS inhibitors target geranylgeranyl diphosphate synthase, blocking the geranylgeranylation of both Rho and Rab proteins and displaying potent anti-tumor effects in MM cells. Overall, IBP inhibitors are novel pre-therapeutic leads for the treatment of MM as activation of the UPR and inhibition of Ras leads to induction of apoptosis and the inhibition of proliferation. Further assessment of combination strategies between IBP inhibitors and bortezomib is warranted. 

Recently recognized as an enabling hallmark of cancer development and progression, the gut microbiome is an area of active investigation [8]. In this Special Issue, Upadhyay Banskota and Skupa et al. [9] summarize key findings in the field concerning the role of microbial populations in lymphomagenesis, diagnosis, and therapy while highlighting the potential for microbiome-directed interventions to enhance therapeutic effects. For instance, nitrogen-recycling bacteria (i.e., Klebsiella and Streptococcus) and the metabolite L-glutamine were highly enriched in MM patients [10]. This enrichment was presumably driven by host-secreted nitrogen (e.g., urea and ammonia) that acted upon the gut microbiota to produce metabolites, contributing to MM progression. In another example, butyrate, a short-chain fatty acid fermented from dietary fiber by gut microbiota, has been shown to elicit anti-proliferative and pro-apoptotic effects [11]. This suggests that the potential use of dietary modifications (i.e., prebiotics) could be used to positively enhance the gut microbiota, providing a protective effect against lymphomagenesis. Additionally, the authors draw attention to the impact of the gut microbiome on responses to immunotherapy through the regulation of TME immune cell function. Studies have shown that prior or concurrent use of antibiotic agents might decrease the treatment efficacy of immune checkpoint inhibitor therapy, which suggests a possible disruption of gut microbial diversity that leads to the impairment of cytotoxic T-cell responses. Overall, Upadhyay Banskota and Skupa et al. emphasize the need for further studies to understand the gut microbiome–immune–oncology axis to provide a more comprehensive understanding of pathogenesis and therapeutic modulation.

Lastly, Munir et al. [12] describe the clinical history of cHL and underline the utility of patient stratification for maintaining balanced maximal survival rates while avoiding treatment-related organ toxicities or acquired secondary malignancies. Furthermore, the authors discuss current and potential therapeutic strategies such as monoclonal antibody-drug conjugates (anti-CD30) immunotherapies (PD-1 checkpoint blockade, CAR T-cell therapy), and small molecule inhibitors (heat shock protein inhibitors, proteasome inhibitors) for patients with high-risk cHL and/or R/R disease. Since CD30 is overexpressed on cHL cells, brentuximab vedotin (anti-CD30 antibody-drug conjugate), in combination with chemotherapy, has recently been added to the FDA-approved treatment repertoire for newly diagnosed high-risk cHL along with immunomodulatory anti-PD-1 checkpoint agents, pembrolizumab and nivolumab, in the setting of R/R cHL [13]. The TME in cHL is made up of regulatory B-cells, T-cells, and NK cells, which release cytokines that aid in their survival and proliferation. A promising therapy for R/R cHL is rituximab (anti-CD20 antibody), which is currently used to treat other B-cell malignancies (e.g., DLBCL, CLL). The use of rituximab in treating cHL is supported by the observations that regulatory B-cells in the cHL TME express CD20. In addition, 20–30% of cHL patients express CD20. Lastly, CD19-directed CAR T-cell therapy (ClinicalTrials.gov identifiers: NCT02348216, NCT02435849) is being evaluated in clinical trials for cHL, despite the lack of CD19 expression on most cHL cells, signifying a need for new CAR T-cell constructs that target CD30. Therapies targeting BCR signaling are currently in preclinical testing with the anticipation of more options being available for R/R patients.

Articles within this Special Issue demonstrate how further investigation into inherent tumor factors/pathways, interactions within the TME, and the gut microbiome–immune–tumor axis could potentially guide the development of clinically useful biomarkers to stratify patients and unveil therapeutically relevant cellular pathways to support the rational design of effective, and safer therapeutic interventions.
